# Predicting drug-target interactions by dual-network integrated logistic matrix factorization

**DOI:** 10.1038/srep40376

**Published:** 2017-01-12

**Authors:** Ming Hao, Stephen H. Bryant, Yanli Wang

**Affiliations:** 1 National Center for Biotechnology Information, National Library of Medicine, National Institutes of Health, Bethesda, MD, 20894, USA

## Abstract

In this work, we propose a dual-network integrated logistic matrix factorization (DNILMF) algorithm to predict potential drug-target interactions (DTI). The prediction procedure consists of four steps: (1) inferring new drug/target profiles and constructing profile kernel matrix; (2) diffusing drug profile kernel matrix with drug structure kernel matrix; (3) diffusing target profile kernel matrix with target sequence kernel matrix; and (4) building DNILMF model and smoothing new drug/target predictions based on their neighbors. We compare our algorithm with the state-of-the-art method based on the benchmark dataset. Results indicate that the DNILMF algorithm outperforms the previously reported approaches in terms of AUPR (area under precision-recall curve) and AUC (area under curve of receiver operating characteristic) based on the 5 trials of 10-fold cross-validation. We conclude that the performance improvement depends on not only the proposed objective function, but also the used nonlinear diffusion technique which is important but under studied in the DTI prediction field. In addition, we also compile a new DTI dataset for increasing the diversity of currently available benchmark datasets. The top prediction results for the new dataset are confirmed by experimental studies or supported by other computational research.

Although enormous research investment and technology advancement have been made in the discovery of new drugs, the number of approved drugs has remained modest. Many promising molecules failed to pass clinical trials due to safety or efficacy issues. As a consequence, there is a pressing need for researchers to identify drug targets and develop effective drugs in innovative ways that could overcome these drawbacks^1^. Drug repositioning, a process of finding new applications for existing drugs, is a potential alternative to new drug discovery, since existing drugs have established clinical and pharmacokinetic data. As reported, many drugs have been successfully identified to be anti-cancer drugs by using repositioning methods. For example, Thalidomide, used earlier as a sedative and anti-emetic agent, was approved by FDA in 1998 for the treatment of erythema nodosum leprosum^2^. Celecoxib was originally developed for the treatment of rheumatoid arthritis and osteoarthritis. Later, it was approved by FDA for the prevention of colon cancer in patients with familial adenomatous polyposis^3^. Rapamycin is an immunosuppressive drug approved in 1999. Some of rapamycin derivatives, such as temsirolimus and everolimus, have received FDA’s approval for the treatment of renal cell cancer and subependymal giant-cell tumors^4^. Many other repurposing drugs have also been tested in Phase I to III clinical trials for various types of cancers^5^,^6^.

It is well known that the synthesis and experimental testing of large number of molecules against drug targets are both costly and time-consuming. Therefore, effective computational approaches have been developed for drug repositioning research, which has proven to be a successful strategy in different fields of in silico drug discovery, such as chemoinformatics^7^,^8^ and structural bioinformatics^9^,^10^. Herein, it should be pointed out that one of the fundamentals for computational drug repositioning is to accurately predict drug-target interactions (DTI). Therefore, researchers in recent years have proposed various computational methods for predicting potential DTI. In 2008, Yamanishi *et al*.^11^ developed a bipartite network method for the integration of chemical and genomic spaces to predict DTI of four classes of targets, i.e., enzymes, ion channels (IC), G protein-coupled receptors (GPCR) and nuclear receptors (NR). Note that since then the dataset they used^11^ has been considered as a golden benchmark by many researchers. Based on this benchmark dataset, several newly developed algorithms showed improved performance. Bleakley *et al*.^12^ proposed a novel supervised inference method to predict unknown drug-target interactions from the benchmark dataset^11^. Results from their kernel-based support vector machine model presented high performance in terms of AUC (area under curve of receiver operating characteristic) and AUPR (area under precision-recall curve). van Laarhoven *et al*.^13^ used a kernel regularized least squares (KRLS) algorithm to predict DTI by solely using the topological information from the adjacency matrix of drug-target network. They defined a Gaussian interaction profile kernel based on the topology profiles. Using this kernel, their model exhibited the significant improvement for AUPR over the state-of-the-art methods at that time. They also found that by combining the topological information with the chemical and genomic information, model performance could be further improved. However, it should be pointed out that the above-mentioned methods were focusing on the setting where both drugs and targets were known, which means that at the stage of building models, each drug or target has at least one known interaction with the corresponding targets or drugs, respectively. In order to extend the methods to the prediction of drugs and targets without any known interaction in the dataset, Mei *et al*.^14^ introduced a neighbor-based interaction-profile inferring method and integrated it into the bipartite local model. As a result, their model performance presented a large improvement. However, the previous kernel-based methods^13^,^14^ only used a simple linear combination technique to form the final kernel matrix from several individual kernels. In fact, such a simple linear setting may not be appropriate when the linear relationship is not evident among kernels. Thus, Hao *et al*.^15^ employed a nonlinear kernel diffusion technique, motivated by the work from Wang *et al*.^16^, to combine different kernels and then using the diffused kernel they adopted KRLS to perform DTI predictions. As a result, the model with the diffused kernel showed better performance than that with the linearly combined kernel. However, when testing with more rigorous validations such as 10-fold cross-validation for the whole dataset, the KRLS algorithm failed to yield satisfied results though it has already adopted an advanced kernel diffusion technique. Recently, Liu *et al*.^17^ proposed a neighborhood regularized logistic matrix factorization (NRLMF) for DTI predictions. The NRLMF model showed an encouraging result based on the 5 trials of 10-fold cross-validation and became the state-of-the-art algorithm in the field. The good performance can be attributed to the following reasons: (1) they took advantage of the merit of logistic matrix factorization, which is especially suitable for binary variables; (2) they proposed an augmented known interaction pairs technique attempting to balance the imbalanced characteristics between known and unknown pairs to some extent; (3) they adopted a neighborhood regularized manner in the objective function; and (4) they used a neighborhood smoothing method to generate new drug/target prediction scores. However, they did not consider the drug-target profile information at all when building the model, which is actually very important for DTI predictions^13^,^14^,^15^. Thus, to integrate the profile information into the model, we propose a four-step procedure for DTI predictions: (1) inferring new drug or target profiles and calculating the profile kernels; (2) diffusing drug kernels; (3) diffusing target kernels; and (4) predicting interaction scores based on the diffused kernels using the proposed algorithm by adding the “trust ensemble” idea into the model. We compare our method to prior arts based on two groups of benchmark datasets. Moreover, we also compile a new DTI dataset on the basis of the latest DrugBank records to enrich the diversity of existing benchmark datasets.

## Material and Methods

### Dataset

Two benchmark datasets were used to validate the proposed algorithm for DTI predictions. One was obtained from the study of Yamanishi *et al*.^11^, which contains the DTI interaction information as retrieved from the KEGG BRITE^18^, BRENDA^19^, SuperTarget^20^ and DrugBank^21^ databases. Protein sequences of targets were obtained from the KEGG GENES database^18^. Chemical compounds were obtained from the KEGG DRUG and COMPOUND databases^18^. The dataset was classified into four groups: enzymes (445 drugs, 664 targets); ion channels (210 drugs, 204 targets); G-protein coupled receptors (223 drugs, 95 targets); and nuclear receptors (54 drugs and 26 targets) as listed in Table 1. Another dataset used in this work was retrieved from the work of Kuang and co-workers^22^. The dataset consists of 3,681 known interaction pairs including 786 drugs and 809 targets (Table 1), whereas: (1) drugs were approved by FDA; (2) drugs included at least one ATC code; and (3) drug structure information was deposited in the KEGG database. Herein, target sequence similarity matrix is denoted by *S*_ts_ (similarity scores among proteins for both datasets were computed using a normalized version of Smith-Waterman score^23^). Chemical structure similarity matrix is denoted by *S*_cs_ (similarity scores among compounds for both datasets were computed using the SIMCOMP tool^24^). The interaction adjacency matrix is denoted by *Y*, where *Y*_ij_ = 1 if drug *i* interacts with target *j*, and *Y*_ij_  =  0 otherwise. The used datasets here are the same as those used in the previous studies^11^,^22^.

### Problem description

**Figure 1:**
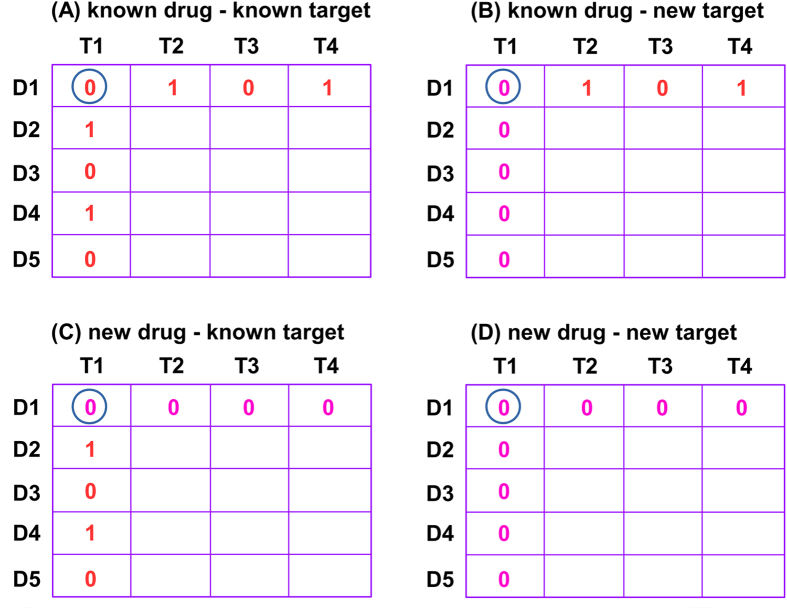
Four scenarios of DTI predictions. For the D1-T1 pair surrounded by circle, (**A**) known drug - known target; (**B**) known drug - new target; (**C**) new drug - known target; and (**D**) new drug - new target.

**Figure 2:**
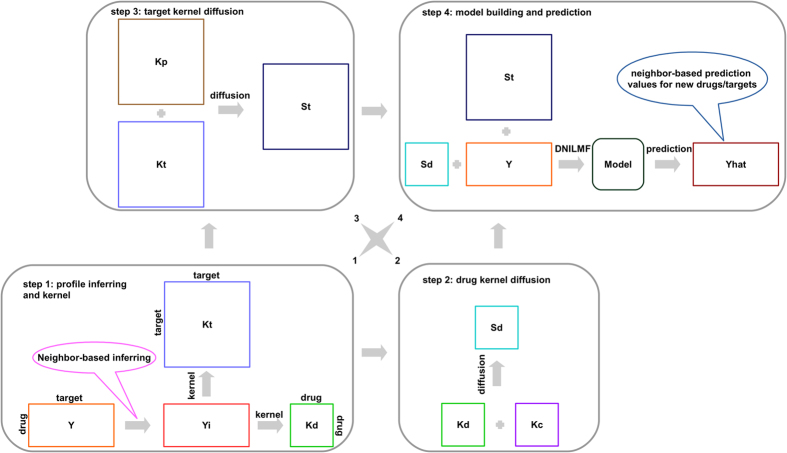
Flowchart of the whole procedure in the proposed DNILMF algorithm.

Given three matrices, *S*_ts_, *S*_cs_ and *Y*, the task is how to make use of them to predict interactions between drug compounds and target proteins, which includes four scenarios (Fig. 1) as described in Hao *et al*.^15^. These scenarios are illustrated by four matrices of 5 drugs (i.e., D1 through D5) and 4 targets (i.e., T1 through T4). Thus, the D1-T1 interaction pair surrounded by circle consists of four cases: (1) known drug - known target (Scenario 1 in Fig. 1A); (2) known drug - new target (Scenario 2 in Fig. 1B); (3) new drug - known target (Scenario 3 in Fig. 1C); and (4) new drug - new target (Scenario 4 in Fig. 1D). Herein, a “known drug” refers to a drug that has at least one interaction with targets (e.g., D1 in Fig. 1A,B, respectively) while a “new drug” refers to a drug that does not have any interaction with targets (e.g., D1 in Fig. 1C,D, respectively) in the dataset. Similar definitions are applied when referring to a “known target” (e.g., T1 in Fig. 1A and C, respectively) and a “new target” (e.g., T1 in Fig. 1B and D, respectively). The goal of this work is to develop a novel algorithm to improve the prediction performance of drug-target interactions. Specifically, the algorithm assigns a score to a drug-target pair estimating the likelihood of an interaction between them, whereas the higher the score is, the more likely the drug and target interact with each other.

### Profile inferring and kernel construction

Though at least one interaction exists for each drug and target in the original benchmark dataset, the scenario of new drug and new target (i.e., Scenario 4) would occur when the dataset is split in the cross-validation process. Thus, the new drug/target interaction profiles were first inferred by its nearest neighbors (number of neighbors, *K*, was set to 5 empirically). The original similarity matrices were converted to kernel matrices (denoted by *K*_c_ and *K*_p_ for the compound (drug) kernel matrix and protein (target) kernel matrix, respectively, see Fig. 2) according to our previous method^15^. Specifically, for a new drug, the inferred drug-target interaction profile was calculated by the multiplication of the chemical similarity of its nearest neighbors with the corresponding drug-target interaction profile. Inferred profiles were normalized at the end by the sum of similarity values between the current drug and its neighbors. Target-drug interaction profile for a new target was calculated in a similar way. Once drugs/targets profiles were inferred for all new drugs and targets (denoted by *Y*i in Fig. 2), the Gaussian kernel matrices were computed, which are denoted by *K*_d_ and *K*_t_ based on the drug profiles and target profiles, respectively.

### Similarity diffusion

Given four kernel matrices, *K*_d_, *K*_c_ for drugs and *K*_t_, *K*_p_ for targets, the goal of the similarity diffusion technique^15^ is to diffuse *K*_d_ and *K*_c_ into one final kernel matrix, *S*_d_, and diffuse *K*_t_ and *K*_p_ into one final kernel matrix, *S*_t_ (see steps 2 and 3 in Fig. 2). The important steps for similarity diffusion are summarized as follows: (1) constructing the “local” similarity matrix for each of the four kernel matrices, which means that given the number of nearest neighbors for the current drug/target (number of nearest neighbors was empirically set to 3), the nearest neighbors were kept while others were set to zeros; (2) diffusing the “local” similarity matrices and the “global” similarity matrices iteratively with a given iteration step number (number of iteration was empirically set to 2) for drugs and targets, respectively. After finishing the iteration process, the status matrices were averaged and normalized to be used as the final diffused matrices (i.e., *S*_d_ for drugs and *S*_t_ for targets). For details of the diffusion procedure, one can refer to the previous studies^15^,^16^.

### Dual-network integrated logistic matrix factorization algorithm

Having obtained the diffused drug similarity matrix *S*_d_ and target similarity matrix *S*_t_, together with the interaction profile matrix *Y*, a dual-network integrated logistic matrix factorization (DNILMF) algorithm was developed for DTI predictions. Herein, a logistic function (i.e., 
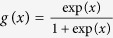
), was used to yield the interaction probabilities between drugs and targets. In NRLMF, *x* = *UV*^*T*^, where *U* and *V* are two latent matrices for drugs and targets, respectively, and *V*^T^ denotes the transpose of *V*. It can be noted that the used logistic function in NRLMF just considered the information from the drug-target interaction network (*Y*) itself. In fact, besides this, the probabilities for predicted interactions may also be influenced by the similarity network information between drugs (*S*_d_) and between targets (*S*_t_). For example, to check if drug D1 interacts with target T1, one intuitive idea is to see if the neighbors of drug D1 interact with target T1, if so, then drug D1 has a higher probability of interacting with target T1. Mathematically, the process can be expressed by *x* = *S*_*d*_*UV*^*T*^. Similarly, if drug D1 interacts with the neighbors of target T1, then there is a higher probability that drug D1 interacts with target T1. Mathematically, it can be expressed by *x* = *UV*^*T*^*S*_*t*_. Actually, a similar idea (“Social Trust Ensemble”) has been proposed in the recommender systems field^25^, which gives the detailed explanation how the similarity network plays a role in the model prediction. Thus, in the current work, the interaction probability scores (ranging from 0 to 1) for drug-target pairs were calculated in the following equation:


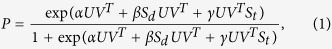


where *α, β, γ* are the corresponding smoothing coefficients with the summation of them as 1 (they were empirically set to *α* = 0.5, and *β* = *γ* = 0.25). Note that equation (1) simultaneously considers both the interaction profile network information (*Y*) and the two similarity network information between drugs (*S*_d_) and targets (*S*_t_). According to the study^17^, by augmenting each known interaction pair to *c* (*c* ≥ 1) folds and by assuming all samples are independent, the probabilities of drug-target interactions were given as follows:


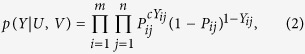


where *c* is the augmented folds for known DTI pairs (*c* was set to 5 empirically). *P*_ij_ refers to the interaction probability between drug *i* and target *j*. The zero-mean spherical Gaussian priors were placed on the drug and target latent vectors as shown in the following equation:


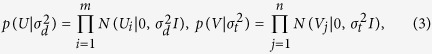


where 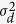 and 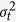 are parameters controlling the variances of Gaussian distributions, *U*_i_ denotes the latent variable for drug *i, V*_j_ denotes the latent variable for target *j* and *I* denotes the identity matrix. Through a Bayesian inference, the following equation was obtained:


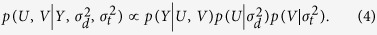


Thus, from the above equations, the log of the posterior distributions for DNILMF were yielded as follows:


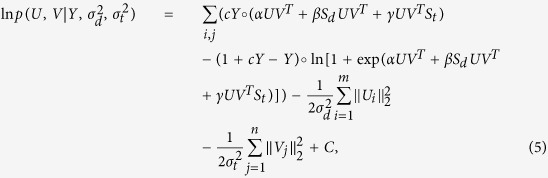


where *C* is a constant which does not depend on the parameters. Thus, two latent variable matrices, *U* and *V*, were generated by maximizing the following objective (log-likelihood, denoted by *LL*) function:


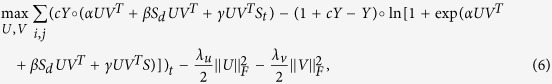


where 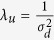, 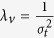, *λ*_*u*_ and *λ*_*v*_ are regularized coefficients for *U* and *V*, respectively (they were empirically set to 5 and 1, respectively), 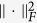 denotes the Frobenius norm, and ° denotes the Hadamard product (element-wise product). Herein, the gradient ascend algorithm was used to solve for *U* and *V* from the above objective function. As a result, the gradient variables for both *U* and *V* were obtained as follows:


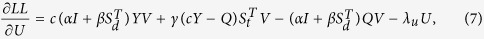



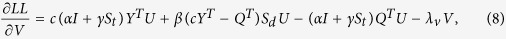


where 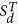, 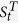, and *Y*^*T*^ denote the transpose of each corresponding matrix, 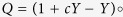

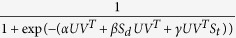
 and *Q*^T^ denotes the transpose of *Q*. In this work, the AdaGrad algorithm^26^ was used to update *U* and *V*. The detailed procedure can be referred to ref. 17.

### Smoothing new drug/target predictions by incorporating neighbor information

As reported in the work^17^, for new drugs/targets, when the drug latent matrix (*U*) and target latent matrix (*V*) were obtained, they were replaced with new ones inferred by using their neighbor information (number of neighbors was empirically set to 5) according to the following equations:


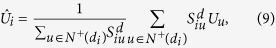



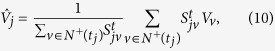


where 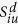 denotes the similarity between a new drug *i* and a known drug *u*; *U*_u_ denotes the latent variable of a known drug *u*. Similar definitions were applied to a new target. Thus, after inferring the latent matrices for new drugs and targets, the predicted interaction probability scores were calculated according to equation (1).

## Results

### Prediction procedure

With the given problem formulation for DTI predictions as described in the method section, we develop a complete algorithm flowchart as shown in Fig. 2. It can be noticed that the prediction procedure includes four steps. The first step is for profile inferring and kernel construction. Given the interaction adjacency matrix *Y*, we first infer the new drug/target profiles (all zeros for the entire row or column in *Y*, which may occur in the cross-validation stage), based on the respective neighbors. The inferred matrix is denoted by *Y*i. At the end of step 1 (see Fig. 2), all the new drug/target profiles are inferred. Based on the complete adjacency profiles (*Y*i), we then calculate the kernels from the drug profiles and target profiles, respectively. Herein, we adopt the Gaussian kernel in the same way as used in our previous work^15^, which results in two kernel matrices, *K*_d_ and *K*_t_ for drug profiles and target profiles, respectively. In the second step, we employ the kernel diffusion method^15^,^16^, an effective but less explored technique in the DTI prediction field, to diffuse two classes of similarity matrices for drugs, *K*_d_ and *K*_c_ (converted from the original compound similarity matrix in the benchmark dataset to the kernel matrix) into one final similarity matrix, denoted by *S*_d_. A similar process is performed for generating the target kernel matrices, *K*_t_ and *K*_p_ (converted from the original protein similarity matrix in the benchmark dataset to the kernel matrix). As a result, a final diffused matrix, *S*_t_, is generated from step 3 as shown in Fig. 2. In step 4, we finally employ our proposed DNILMF (dual-network integrated logistic matrix factorization) algorithm to perform DTI predictions. It should be pointed out that, in this last step, new drug/target interaction scores are re-computed based on their neighbor prediction values instead of their own values generated directly by the model. Our source code is available at: https://github.com/minghao2016/DNILMF.

### Comparison with the state-of-the-art algorithms

To validate DNILMF, we compare our results with those from the state-of-the-art algorithms. Firstly, we compare the DNILMF algorithm with NRLMF which previously achieved the best performance based on the benchmark dataset proposed by Yamanishi and co-workers^11^. Using the same dataset and similar cross-validation methods (i.e., 5 trials of 10-fold cross-validation under three settings: (1) CVP, cross-validation based on the drug-target pairs (see Fig. 1A); (2) CVR, cross-validation based on the rows (see Fig. 1C); and (3) CVC, cross-validation based on the columns (see Fig. 1B)), and for all of the four sub-groups, our proposed DNILMF algorithm outperforms NRLMF in terms of both AUC and AUPR, especially for AUPR as shown in Tables 2, 3, 4. In fact, all of the four sub-groups in the benchmark dataset possess the imbalanced characteristics, which means that the number of drug-target pairs with known interactions is far less than the number of pairs with no interaction evidence. Therefore, a more sensitive AUPR metric is generally preferred for assessing the prediction results for those imbalanced datasets. It can also be noted that DNILMF outperforms NRLMF with larger ratios of AUPR (i.e., AUPR1/AUPR2) than AUC (i.e., AUC1/AUC2), indicating DNILMF exhibits a stronger power for handling highly imbalanced datasets. In particular, it is interesting to note that for the GPCR class in Table 2, DNILMF outperforms NRLMF by over 6% in terms of AUPR under the setting CVP, indicating that DNILMF is more powerful to predict interactions between ligands and the target class of membrane proteins using the ligand-based method. Thus, DNILMF provides a complementary technology for the receptor-based methods (such as docking), which experience more challenges when applied to the GPCR class since the 3D crystal structures for membrane proteins are difficult to obtain. Under the setting CVR (i.e., new drugs, see Fig. 1C), DNILMF largely outperforms NRLMF indicating it can handle the new drug scenario better than NRLMF (see Table 3). Under the setting CVC (i.e., new targets, see Fig. 1B), DNILMF also consistently outperforms NRLMF (see Table 4). By comparing various settings for DTI predictions, it is evident that CVP is the easiest case for DNILMF, since more known information is available to train a model compared to the settings of CVR and CVC. It can also be noted that for the datasets with more samples (e.g., Enzymes and IC), the AUPR and AUC metrics from CVC in DNILMF are better than those from CVR. By contrast, for the datasets with less samples (e.g., GPCR and NR), the AUPR and AUC values from CVR are better than those from CVC. The phenomenon can basically be confirmed by NRLMF except that for the GPCR dataset, NRLMF presents better AUPR and AUC values from CVC than those from CVR. Under the settings of CVR and CVC, the decreased performance is due to the fact that there exists less known information in the training phase and the obtained latent variables for new drugs/targets may not be accurate^17^. Among the four types of scenarios, the most difficult case for DTI predictions is Scenario 4 (i.e., new drug - new target, see Fig. 1D), which may be generated during cross-validation. Taking the setting CVP as an example, in the course of cross-validation whereas datasets of training and testing are re-generated by a randomized procedure, samples of new drugs and targets may be left in the testing dataset so that the drug-target pairs fall into the new drug - new target category (see the D1-T1 pair in Fig. 1D). We compare DNILMF with NRLMF (it is derived from our implementation using the R software^27^, which is slightly different from the original one) in such a difficult case. We take the GPCR data under the setting CVP as an example and run 5 times of “5 trials of 10-fold cross-validation”. As a result, DNILMF gives AUPR of 0.633 ± 0.025 and AUC of 0.897 ± 0.004, while NRLMF exhibits AUPR of 0.385 ± 0.006 and AUC of 0.706 ± 0.008 indicating that DNILMF has an advantage in making DTI predictions for new drug - new target pairs over NRLMF. The results are obtained based on the default parameters for both algorithms. Our source code shows the detailed process. We argue that the better performance may benefit from the diffused kernels. To validate this, we plug the diffused kernels into another popular DTI prediction algorithm, KBMF^28^. We run KBMF with the default parameters except that the number of latent variables is set to 20. We take the NR data as an example for computational consideration and run the algorithm, under the setting CVP with 5 trials of 10-fold cross-validation, KBMF gives AUPR of 0.514 ± 0.026 and AUC of 0.883 ± 0.012 when using similarity matrices just from the structure information (i.e., *K*_c_ and *K*_p_ in steps 2 and 3 shown in Fig. 2). When plugging the diffused kernels (i.e., *S*_d_ and *S*_t_ in steps 2 and 3 shown in Fig. 2), KBMF gives AUPR of 0.643 ± 0.017 and AUC of 0.919 ± 0.012. Undoubtedly, the diffused kernels play a critical role in the performance improvement for KBMF. The detailed comparison is given in our source code.

Besides testing with the commonly used benchmark dataset, we also validate our algorithm with an additional benchmark dataset compiled by Kuang *et al*.^22^, which is a larger dataset with 3,681 known interactions including 786 drugs and 809 targets used together in an eigenvalues transformation technique (denoted by EigenTrans) to boost the prediction accuracy of DTI. As shown in Table 5, our algorithm outperforms EigenTrans by around 2% in terms of AUC, and more significantly by 10% in terms of AUPR based on the setting CVP as used in EigenTrans. In summary, the proposed DNILMF algorithm shows better performance in comparison to the state-of-the-art approaches based on the benchmark datasets under the all four types of scenarios.

### Influence of parameters

It should be pointed out that all obtained DNILMF results described above are based on the empirical setting of parameters. However, the optimal performance of most algorithms depends on the parameter settings. Thus, we vary six parameters and investigate their influence on the performance of DNILMF. The number of latent variables (*numLatent*) is changed from 30 to 100 incremented by 10 at a step. The augmented number for known interaction pairs (*c*) is changed from 3 to 10 incremented by 1 at a step. The coefficient of latent matrix product, *α*, is changed from 0 to 1 incremented by 0.1 at a step. The *λ*_*u*_ and *λ*_*v*_, regularized coefficients of latent variables for drugs and targets, are changed from 1 to 10 incremented by 1 at a step, respectively. The number of neighbors (*K*) for inferring new drug/target profiles and smoothing new drug/target predictions is changed from 1 to 10 incremented by 1 at a step. Herein, we only change one parameter at a time while fixing others at the default parameters (i.e., *numLatent* = 50, *c* = 5, *α* = 0.5, *λ*_*u*_ = 5, *λ*_*v*_ = 1, *K* = 5). Thus, under the setting CVP and taking the GPCR data as an example, we finally obtain AUPR of 0.853 and AUC of 0.979 based on the optimal parameters (i.e., *numLatent* = 90, *c* = 6, *α* = 0.4, *λ*_*u*_ = 2, *λ*_*v*_ = 2, *K* = 2). Evidently, the tuned parameters boost the performance of DNILMF comparing to the results from the default parameters, i.e., AUPR of 0.812 and AUC of 0.975. It should also be emphasized that if one explores the parameter space largely using techniques such as genetic algorithm, the model performance and efficiency of hyper-parameter optimization may be further improved. However, it is worthwhile to point out that, even without parameter optimization, the obtained results have already exhibited better performance than those from the state-of-the-art algorithms, which is the reason that we take the quicker path for parameter tuning rather than taking the approach for an exhaustive search to explore the entire parameter space and the utmost optimal combination.

### Prediction and validation of new compiled DTI dataset

To enhance the diversity of benchmark datasets and facilitate more rigorous assessment for DTI prediction algorithms, we have compiled a new DTI dataset with PubChem CID identifier for drugs and UniProt identifier for targets. First, we obtain the mapping (denoted by CID-DBID) for CID (PubChem Compound ID) and DBID (DrugBank drug ID) from PubChem (https://pubchem.ncbi.nlm.nih.gov/), publicly available biological and chemical information database, and manually inspect the obtained file to make sure that all the CID-DBID mappings are on the one to one basis. We then extract the approved drug-target interaction information from DrugBank^21^ (released on April 20 2016) and we only keep the small molecule drugs which are mapped to CID. For the protein sequence file, we use the FASTA format of sequences provided by DrugBank, which are approved target polypeptide sequences (released on April 20 2016). We keep the sequences of *Homo sapiens* only and have the obsoleted ones removed. At this point, a total of 5,249 known drug-target interactions annotated by DrugBank are obtained. A few filters are applied subsequently to the drug molecules for the consideration of data consistency, including removing mixture drugs and drug molecules with molecule weight falling out of the range of 150 to 500 Dalton. For the target sequences, we keep those with the number of amino acids in the range of 100 to 900. Several duplicated interactions (e.g., interactions from the same CID and target sequence pair) are also removed. Finally, a new dataset is compiled with 3,688 known interactions consisting of 829 unique drugs and 733 unique targets, which is summarized together with the other two benchmark datasets in Table 1, and the detailed information for the new compiled dataset is provided in the Supplementary Dataset. A sparsity value (known interactions divided by all possible interaction pairs) is calculated for each dataset. From Table 1, one can notice that the dataset from Yamanishi *et al*.^11^ has higher sparsity values due to that the targets are classified into four sub-groups. On the contrary, the datasets from Kuang *et al*.^22^ and ours use the interaction information from DrugBank as a whole without sub-setting, which leads to a lower sparsity value (0.006 for both, see Table 1). In fact, a sparser dataset (with lower sparsity values) will make the prediction more challenging. Based on the new compiled dataset, we apply our proposed algorithm (all parameters are fixed to the default values) to perform DTI predictions. Herein, we calculate the similarity matrix for drugs using the Tanimoto coefficient based on two classes of fingerprints (PubChem fingerprint (denoted by pcfp) and a path-based fingerprint (denoted by fp2)) using the R software^27^,^29^,^30^. For targets, we also obtain two kinds of similarity matrices based on the clustal omega software (denoted by clusto)^31^ and the spectrum kernel (denoted by kmer3, one parameter *kmers* is set to 3)^32^. It should be pointed out that clusto generates distant matrix (denoted by distM), and the result of (1 - distM) is calculated to obtain the corresponding similarity matrix. Thus, four classes of combinations (i.e., fp2-clusto, fp2-kmer3, pcfp-clusto and pcfp-kmer3) are formed for testing the algorithm based on the setting CVP, and the respective results obtained are shown in Table 6. It can be noted that despite of the extreme sparsity of the dataset, the model performance is still satisfactory with AUC and AUPR of more than 0.970 and 0.772, respectively. Among them, the pcfp-kermer3 combination gives the best result with AUC of 0.972 and AUPR of 0.775. Our previous study also showed that the pcfp-kmer3 combination generated the better results^15^.

Since the DNILMF algorithm combined with pcfp-kmer3 gives the best results for the new compiled dataset, we in the following take this test as an example to further analyze the results in a greater detail by looking into the novel predictions. Table 7 lists the top 5 predicted interactions (i.e., interactions not indicated in the new compiled dataset) sorted in descending order of the prediction scores. The top one predicted interaction occurs between DB00370 (Mirtazapine) and P08908 (5-hydroxytryptamine receptor 1A, 5HR1A), a membrane protein, with a prediction score of 0.921. Mirtazapine, with a tetracyclic chemical structure, is an antidepressant used for the treatment of moderate to severe depression. Originally, Mirtazapine interacts with 22 targets as reported in the DrugBank database (see Supplementary Dataset). Here, the DNILMF algorithm predicts that it may also interact with 5-hydroxytryptamine receptor 1A (5HR1A). To validate the predicted interaction between Mirtazapine and 5HR1A, we search PubChem using this drug (CID 4205) and notice that PubChem BioAssay ID (AID) 438555 derived from the in-silico work of Langham *et al*.^33^ reports a positive result regarding Mirtazapine’s binding with 5HR1A. The second top prediction with a score of 0.906 is formed between Flunitrazepam (DB01544) and Gamma-aminobutyric acid receptor subunit alpha-1 (GARSA1, ion channel). Flunitrazepam consists of a benzodiazepine with pharmacologic actions similar to diazepam that can cause anterograde amnesia. Due to the fact that it may precipitate violent behavior, the US government has banned the importation of this drug. Having six known interactions in the compiled dataset, it is predicted to form interaction with another target, GARSA1. In fact, the prediction can be supported by the experimental result from Collins and co-workers^34^ with data reported in PubChem BioAssay AID 72640. DB0036 (Clozapine) interacts with 26 targets as reported in the DrugBank database. Herein, it is predicted to interact with Dopamine D5 receptor (DD5R), a member of the GPCR 1 family, with a score of 0.903. The experimental study^35^ and the data in PubChem AID 392466 confirm our prediction. The fourth predicted interaction occurs between Methysergide (DB00247) and 5-hydroxytryptamine receptor 1D (5HR1D), a GPCR 1 family. Methysergide is used prophylactically in migraine and other vascular headaches and used to antagonize serotonin in the carcinoid syndrome, which forms 8 interactions with targets in the compiled dataset. Our prediction is supported by the result reported in T3DB^36^ (T3D2726). Loxapine, an antipsychotic agent used in schizophrenia, forms 32 interactions in the compiled dataset. It is predicted to interact with 5-hydroxytryptamine receptor 2B (5HR2B), a GPCR 1 family. The prediction result is consistent with the study by Alaimo and co-workers^37^ that ranked the prediction score between Loxapine and 5HR2B at the seventh position out of all 117 pairs.

## Discussion

Various methods have been proposed to perform DTI predictions such as similarity-based methods, conventional machine learning methods as well as matrix factorization-based methods. Among them, MF (matrix factorization)-based ones have shown the best prediction accuracy according to the recently reported work by Liu and co-workers^17^. They used the neighborhood regularized logistic matrix factorization (NRLMF) approach to perform DTI predictions based on the benchmark dataset^11^. The strength of NRLMF is contributed by: (1) the logistic function used; (2) the augmented known DTI pairs; (3) neighbor-based regularization; and (4) neighbor-based inference at the prediction step. In this work, we first take advantage of some of the strength in NRLMF. We also re-formulate the objective function by adding the network regularization into the logistic function to determine the predicted scores. More importantly, we employ the nonlinear diffusion technique among similarity matrices, which is less exploited in the past except in our recent work^15^. As a result, predictions are significantly improved. The underlying idea in our proposed objective function lies in the fact that similar drugs (or targets) may contribute to the accuracy of the predictions for their neighbors. In fact, the recommender systems based on the social networks have proposed the idea called “Social Trust Ensemble”^25^. Indeed, progress for one field may be accelerated by “borrowing” ideas, concepts or theories from a different discipline. To the best of our knowledge, it is the first time to incorporate the “Trust Ensemble” idea to the drug-target prediction subject in this work. It is worthy of mentioning that strategy development for constructing metrics to boost the model performance is an important research subject to be studied in different fields such as neural image^38^. Two categories of combination methods are often used to obtain ultimately learned metrics with better prediction ability. One is derived from supervised multiple kernel learning, and the other is unsupervised learning. The latter one is easier and flexible to combine with other algorithms since it can be obtained before the model building step, while the former should be integrated with the model learning process. Thus, unsupervised algorithms are often adopted by researchers in the medicinal and computational chemistry fields due to the simplicity and easy implementation^15^. In the previous studies of DTI predictions, a simple linear combination of multiple similarity matrices was often used. Although the combination improved the prediction accuracy compared to those models derived from a single similarity matrix just based on the structure/sequence information, we argue that such a simple linear combination may not always be appropriate due to the possible nonlinear relationship among the similarity metrics. Thus, nonlinear combination technologies should be employed to extract the proper information from different metrics. Kernel diffusion is one of the nonlinear techniques to effectively extract and combine the rich information in different similarity metrics, which augments the usage of the most important information while suppressing the signal from the least useful information through a complementary diffusion process^16^. The technique has been successfully applied to various fields such as genomic research^16^. However, little attention is paid to this advanced technique in the DTI prediction area except in a previous work from our group^15^. Thus, to further explore the technique, we employ the nonlinear diffusion procedures in this work to combine similarity matrices for drugs and targets leading to the final and optimized matrices which contain the most powerful information. Such a nonlinear diffusion technique has proven to play a critical role in improving the model performance. It should also be pointed out that the neighbor information based post-processing of prediction scores for the new drugs/targets, which also takes advantage of the diffused similarity matrices, is also important for model performance enhancement. In summary, a new algorithm, DNILMF, is developed with improved DTI predictions in comparison to the previous studies. The gained performance in the current work is contributed not only by the proposed dual-network integrated logistic matrix factorization function, but also, and even more importantly, by the advanced nonlinear diffusion technique. Therefore, we hope that the nonlinear combination technique can be extensively explored in the DTI prediction field, and we plan to explore other diffusion algorithms in future work with the adapted weight for different similarity metrics. We also compile a new dataset to increase the diversity of benchmark datasets in the field. We believe the current work will increase research productivity toward drug repositioning and polypharmacology.

## Additional Information

**How to cite this article**: Hao, M. *et al*. Predicting drug-target interactions by dual-network integrated logistic matrix factorization. *Sci. Rep.* **7**, 40376; doi: 10.1038/srep40376 (2017).

**Publisher's note:** Springer Nature remains neutral with regard to jurisdictional claims in published maps and institutional affiliations.

## Supplementary Material

Supplementary Dataset 1

## Figures and Tables

**Table 1:** Summary of our compiled dataset, and the other two benchmark datasets.

		Number of drugs	Number of targets	Number of interactions	Sparsity
Yamanishi	Enzymes	445	664	2,926	0.010
	IC	210	204	1,476	0.034
	GPCR	223	95	635	0.030
	NR	54	26	90	0.064
Kuang	—	786	809	3,681	0.006
Hao	—	829	733	3,688	0.006

**Table 2:** The comparison of DNILMF with NRLMF using 5 trials of 10-fold cross-validation based on the setting CVP.

Data	Method	AUPR	AUC
Enzymes	NRLMF	0.892 ± 0.006	0.987 ± 0.001
	DNILMF	0.922 ± 0.008	0.989 ± 0.001
IC	NRLMF	0.906 ± 0.008	0.989 ± 0.001
	DNILMF	0.938 ± 0.008	0.990 ± 0.001
GPCR	NRLMF	0.749 ± 0.015	0.969 ± 0.004
	DNILMF	0.812 ± 0.009	0.975 ± 0.003
NR	NRLMF	0.728 ± 0.041	0.950 ± 0.011
	DNILMF	0.751 ± 0.031	0.955 ± 0.004

**Table 3:** The comparison of DNILMF with NRLMF using 5 trials of 10-fold cross-validation based on the setting CVR.

Data	Method	AUPR	AUC
Enzymes	NRLMF	0.358 ± 0.040	0.871 ± 0.017
	DNILMF	0.796 ± 0.029	0.964 ± 0.009
IC	NRLMF	0.344 ± 0.033	0.813 ± 0.027
	DNILMF	0.822 ± 0.047	0.961 ± 0.010
GPCR	NRLMF	0.364 ± 0.023	0.895 ± 0.011
	DNILMF	0.781 ± 0.050	0.967 ± 0.006
NR	NRLMF	0.545 ± 0.054	0.900 ± 0.021
	DNILMF	0.776 ± 0.026	0.956 ± 0.010

**Table 4:** The comparison of DNILMF with NRLMF using 5 trials of 10-fold cross-validation based on the setting CVC.

Data	Method	AUPR	AUC
Enzymes	NRLMF	0.812 ± 0.018	0.966 ± 0.005
	DNILMF	0.889 ± 0.023	0.978 ± 005
IC	NRLMF	0.785 ± 0.028	0.964 ± 0.007
	DNILMF	0.887 ± 0.010	0.970 ± 0.004
GPCR	NRLMF	0.556 ± 0.038	0.930 ± 0.012
	DNILMF	0.684 ± 0.036	0.933 ± 0.009
NR	NRLMF	0.449 ± 0.079	0.851 ± 0.027
	DNILMF	0.483 ± 0.050	0.856 ± 0.042

**Table 5:** The comparison of DNILMF with EigenTrans using 5 trials of 10-fold cross-validation based on the setting CVP.

Method	AUPR	AUC
EigenTrans	0.649 ± 0.034	0.941 ± 0.005
DNILMF	0.748 ± 0.009	0.965 ± 0.001

**Table 6:** Prediction results of DNILMF for the new compiled dataset.

Combination	AUPR	AUC
fp2-clusto	0.772 ± 0.011	0.970 ± 0.001
fp2-kmer3	0.772 ± 0.010	0.970 ± 0.001
pcfp-clusto	0.774 ± 0.009	0.971 ± 0.001
pcfp-kmer3	0.775 ± 0.011	0.972 ± 0.001

**Table 7:** Top 5 predicted interactions for the new compiled dataset using DNILMF based on the pcfp-kmer3 combination.

Rank	CID	DrugBank ID	Drug name	UniProt ID	Target name	Score
1	4205	DB00370	Mirtazapine	P08908	5HR1A	0.921
2	3380	DB01544	Flunitrazepam	P14867	GARSA1	0.906
3	2818	DB00363	Clozapine	P21918	DD5R	0.903
4	6540428	DB00247	Methysergide	P28221	5HR1D	0.898
5	3964	DB00408	Loxapine	P41595	5HR2B	0.892
